# Survival outcomes of marijuana users in p16 positive oropharynx cancer patients

**DOI:** 10.1186/s40463-019-0365-4

**Published:** 2019-09-02

**Authors:** Han Zhang, Michael Xie, Marc Levin, Stuart D. Archibald, B. Stanley Jackson, J. E. M. Young, Michael K. Gupta

**Affiliations:** 1 Division of Otolaryngology—Head and Neck Surgery, McMaster University, Hamilton, Ontario Canada; 2 Michael DeGroote Faculty of Medicine, McMaster University, Hamilton, Ontario Canada

**Keywords:** Marijuana, p16 positive oropharyngeal cancer, Survival outcomes

## Abstract

**Background:**

Oropharynx squamous cell carcinoma (OPSCC) has become the predominant subsite for head and neck mucosal cancers (HNC) due to the rise of human papillomavirus (HPV) related disease. Previous studies have suggested an association between marijuana use and HPV-related OPSCC. Despite this, no study has examined the potential relationship between marijuana use and survival in this subset of patients.

**Objective:**

To examine the survival outcomes of HPV-related OPSCC patients in marijuana users.

**Methods:**

Patients who were marijuana users were identified from a prospectively collected database of HNC patients from January 2011 to 2015. A physical review of clinic records was undertaken to extract relevant patient, tumor, treatment, follow-up, as well as survival data. Patients greater than 17 years of age with pathologically proven p16 positive OPSCC were included. They were then case-matched in a 1-to-1 basis to patients who were non-marijuana users based on age, gender, and cTNM staging.

**Results:**

Forty-Seven patients met inclusion criteria within each group. Univariate logistic regression analysis showed that age, gender, and cT-Stage were predictive of disease recurrence within both groups (*p* < 0.05). However, cN-stage, treatment modality, tumor subsite, tobacco use, and tobacco dose were not (*p* > 0.05). There was no statistically significant difference between marijuana and non-marijuana user groups in 5-year (*p* = 0.400) overall survival, disease-specific (*p* = 0.993), disease-free (*p* = 0.404), and metastasis-free survival (*p* = 0.384).

**Conclusions:**

No survival difference is found between HPV-related OPSCC marijuana users and non-users. This finding has implications for both de-escalation regimes and the use of cannabis as a therapeutic agent.

## Introduction

Oropharyngeal squamous cell carcinoma (OPSCC) has become the predominant subsite for head and neck mucosal cancers (HNC) largely attributed to increased acquisition of oral human papillomavirus (HPV) through changes in sexual behavior [1]. A significant increase within the HPV positive disease population is especially seen within the < 45 years age group [2, 3]. Different mechanisms of carcinogenesis coupled with separate molecular profiles between HPV positive and negative OPSCC have created two distinctive diseases with varied outcomes [4–6]. As a result of the improved treatment response of HPV positive OPSCC [7–10], a move to de-escalate treatment within this unique patient population has been implemented as a method of reducing long-term treatment toxicities and side-effects [11–14]. In order to de-intensify treatment regimens, further prognostic factors are still needed to help sub-classify patients that might be candidates.

*Cannabis sativa*, otherwise known as marijuana, is one of the most commonly used illicit substance where the life-time prevalence of use is reported to be as high as 42.5% [15]. Canada’s imminent legalization of the recreational use of marijuana may well increase this already sizeable number. Marijuana with its known anti-anxiety, anti-emetic, analgesic, as well as anti-depression effects has recently shown promise in HNC as an adjunct in treating psychosocial as well as quality of life issues [16]. However due to studies that have hypothesized the link between marijuana and HPV positive OPSCC, the use of marijuana as a treatment to improve general well-being amongst HNC has been thought of with hesitation. A pooled analysis from the INHANCE Consortium as well as a case-controlled study did indeed show a strong association between HPV-positive OPSCC and marijuana use recently [7, 17]. However, the association between marijuana and OPSCC remains uncertain. It is unclear whether marijuana use is primarily oncogenic on its own or if marijuana use is associated with secondary factors, such as increased sexual promiscuity, which increase the risk of OPSCC. .

Given the need to consider de-escalation regimens for OPSCC and the unclear link between marijuana and OPSCC, we sought to study the effects of marijuana use on survival outcomes of HPV-positive OPSCC patients.

## Methods

The study was fully approved by the Hamilton Integrated Research Ethics Board.

### Patients and data collection

Patients were enrolled consecutively and prospectively at the time of their HNC diagnosis into a database. All patients were treated at a single tertiary care cancer center and were enrolled from January 2011 to January 2015. Patient demographics, comorbidities, smoking status (never vs ever), and lifetime tobacco exposure (≤ 10 vs > 10 pack years), marijuana use, tumor characteristics, and treatment regimens were all collected prospectively as part of the database. A physical review of outpatient, inpatient, and cancer clinic records was undertaken to confirm data accuracy and extract relevant patient, tumor, treatment, follow-up, as well as survival data. Age adjusted Charlson Comorbidity Index (CCI) scores were calculated using relevant comorbidities [18]. Follow-up data up to June 2018 was collected within the chart review.

Patients who were current marijuana users were self-identified from the database and were confirmed to be ≥ 17 years of age, have pathologically diagnosed p16 positive oropharynx squamous cell carcinoma treated with curative intent. These patients were then case-matched to patients who were non-marijuana users from the database in a 1-to-1 scheme based on age, gender, and clinical TNM-Staging. All patients who were included within the non-marijuana user group met the same inclusion criteria as those within the marijuana user group. Marijuana use was defined as current loose-leaf usage on an at least weekly basis. All patients within the marijuana user group were using marijuana at the time of the data collection.

### Outcomes

The primary outcome measure was set as overall survival. This was calculated as the time from the first date of treatment to the date of death or date the patient was last known alive according to electronic medical records and/or clinic follow-up records. Secondary outcomes included disease recurrence, disease-specific, and disease-free survival. Disease recurrence was defined as frequency of first detected recurrence of OPSCC (local, regional or distant. Disease-specific survival was defined as the time from the first day of treatment to death as a result of the primary cancer. Disease-free survival was calculated from the first day of treatment to the date of disease recurrence anywhere in the body. Metastasis-free survival was defined as the time from the first day of treatment to the date of distant metastasis detection.

### Staging

Staging of the tumours was clinical and according to the seventh edition of the American Joint Committee on Cancer (AJCC) TNM staging manual (2009).

### Treatment

All patients had their treatments reviewed and decided by a multi-disciplinary HNC treatment team. Depending on the patient and tumor characteristics, treatment was either prescribed as primary radiotherapy, or primary chemoradiotherapy (CRT), or primary surgery. Surgery for all patients within the study was done via a transoral robotic approach (TORS). Patients who were staged cT1–2, N0-2a were deemed candidates for TORS. Adjuvant radiotherapy was prescribed for patients with close margins and or greater than N2a disease and or extranodal extension on final pathology.

### Follow-up

All patients were followed at tertiary cancer treatment center at regular intervals following treatment. Dates of follow-up up to June 2018 were recorded. Patients who were suspected of disease recurrence were investigated by biopsy and full metastatic work-up.

### Statistical analysis

Baseline characteristics were compared using standard modes of comparison between multiple groups. Continuous data was analyzed using ANOVA, with Bonferoni correction. Categorical data was compared using the chi-squared test. Overall, disease-specific, disease-free and metastases-free survival analyses were performed using Kaplan-Meier analyses to determine year-specific estimated actuarial survival rates. The log-rank test was employed to determine the presence of significant differences (*p* < 0.05) between different treatment groups. Univariate analysis was done to assess for predictors of disease recurrence. Patients were grouped by marijuana use. Analyses were performed with SPSS Statistics 19.0 (SPSS Inc., Chicago, IL).

## Results

### Patient characteristics

Patient characteristics are shown in Table 1. Forty-Seven patients were identified as marijuana users who met inclusion criteria. These patients were then case matched to 47 non-marijuana users in a 1-to-1 fashion. The mean age for the marijuana user group was 60.6 years (range: 40.3–85.9 years) while the mean age for the non-marijuana user group was 60.4 years (range: 40.8–84.8 years). Both groups had predominantly male populations. There was no statistically significant difference in mean age (*p* = 0.718) and gender (*p* = 1.000) between the two groups. The mean Karnofsky score was found to be 92.3 for the marijuana and 91.7 for the non-marijuana group (*p* = 0.967) and the mean CCI-score was found to be 1.1 for the marijuana and 1.2 for the non-marijuana group (*p* = 0.974). There was also no statistically significant difference in tobacco use between marijuana users and non-users (*p* = 0.914), however marijuana users who also used tobacco were more likely to have ≤ 20 pack year history of use as compared to non-marijuana users (74.3 vs 63.9, *p* = 0.041).

**Table 1:** Patient demographics

Variable	Non-Marijuana user	Marijuana user	*p*-Value
N	47	47	
Age			0.718
Mean, yrs	60.4	60.6	
Range, yrs	40.8–84.8	40.3–85.9	
Gender, no. (%)			1.000
Female	9 (19.1)	9 (19.1)	
Male	38 (80.9)	38 (80.9)	
Tobacco Use			0.914
Never	36 (76.6)	35 (74.5)	
Ever	11 (23.4)	12 (25.5)	
Tobacco Dose			0.041
≤ 20 pack year	23 (63.9)	26 (74.3)	
> 20 pack year	13 (36.1)	9 (25.7)	
Charlson Comorbidity Index, Mean (Range)	1.2 (1.0–2.1)	1.1 (1.0–2.2)	0.974
Karnofsky Score, Mean (Range)	91.7 (70–100)	92.3 (65–100)	0.967

Table 2 shows tumor characteristics. Tonsil was the most common subsite (*n* = 37, 78.7%). Most patients presented with T2 disease within both the marijuana user (*n* = 20, 42.6%) and non-user (*n* = 22, 46.8%) groups. Most patients also presented with N2a nodal burden within the marijuana user (*n* = 19, 40.4%) and non-user (*n* = 17, 36.2%) groups. Both groups of patients had CRT as the primary mode of treatment (*n* = 25 and 26, 53.2 and 55.3%). There were no statistical significant differences in tumor subsite, T and N stage, and treatment modality between the two groups (*p* > 0.05).

**Table 2:** Tumor characteristics

Variable	Non-Marijuana user	Marijuana user	p-Value
Tumor Subsite, no. (%)			0.293
Tonsil	37 (78.7)	35 (74.5)	
Base of Tongue	9 (19.1)	10 (21.3)	
Other	1 (2.1)	2 (4.3)	
cT-Stage, no. (%)			0.388
1	11 (23.4)	11 (23.4)	
2	20 (42.6)	22 (46.8)	
3	10 (21.3)	10 (21.3)	
4	6 (12.8)	4 (8.5)	
cN-Stage, no. (%)			0.835
0	11(23.4)	11 (23.4)	
1	8 (17.0)	8 (17.0)	
2a	19 (40.4)	17 (36.2)	
2b	5 (10.6)	7 (14.9)	
2c	2 (4.3)	2 (4.3)	
3	2 (4.3)	2 (4.3)	
Primary Treatment			0.978
S	5 (10.6)	4 (8.5)	
S-RT	1 (2.1)	2 (4.3)	
RT	16 (34.0)	15 (31.9)	
CRT	25 (53.2)	26 (55.3)	

Abbreviations: *S* Surgery, *RT* Radiotherapy, *C* Chemotherapy

### Disease recurrence

Of the 47 patients within the marijuana user group, recurrence occurred in 5 (10.5%) patients. The site of recurrence was local in 2 (4.2%), regional in 1 (2.1%), and distant in 2 (4.2%) patients. Patients within the non-marijuana user group had recurrence occurring in 6 (12.3%) patients. The site of recurrence was local in 2 (4.2%), regional in 1 (2.1%), and distant in 3 (6.3%) patients. Univariate logistic regression analysis showed that age, gender, and cT-Stage were predictive of disease recurrence within both groups (*p* < 0.05). However, cN-stage, treatment modality, tumor subsite, tobacco use, and tobacco dose were not predictive of disease recurrence within both groups (*p* > 0.05).

### Survival outcomes

The mean follow-up for all patients was 4.12 years (SD = 2.3 years, Range = 2.52–7.25 years).

**Fig. 1:**
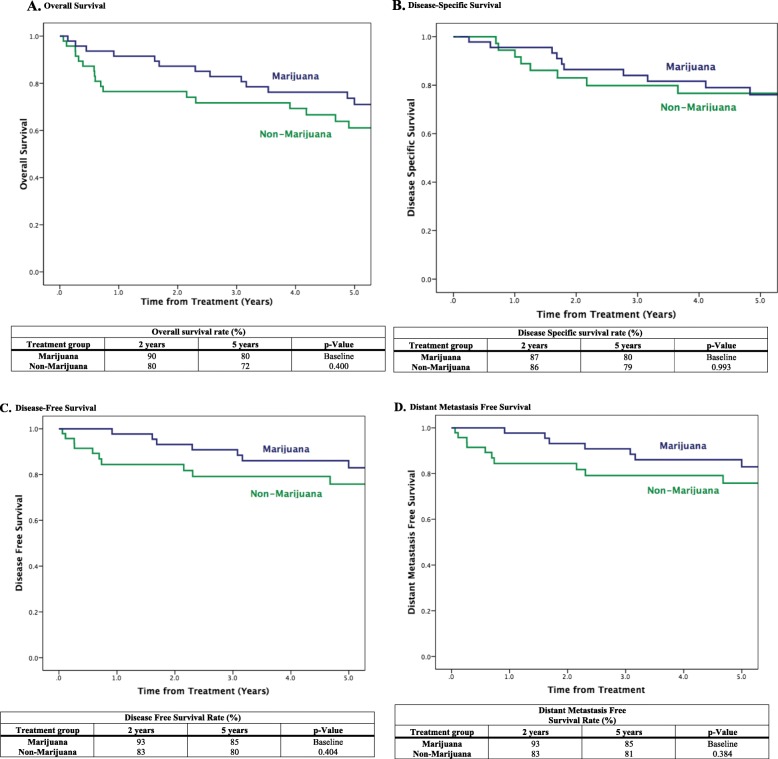
Survival Outcomes. **a**. Overall Survival. **b**. Disease-Specific Survival. **c**. Disease-Free Survival. **d**. Distant Metastasis Free Survival

A total of 5 (10.6%) patients died from the marijuana user and non-marijuana user group. Within both groups, 3 (6.4%) patient died as a result of their cancer and 2 (4.2%) from other causes. Two and five-year overall survival are shown in Fig. 1a. There was no statistically significant difference (*p* = 0.400) between marijuana and non-marijuana user groups in 2-year (90 vs 80%) and 5-year (80 vs 72%) survival. Similar results were found for 5-year disease-specific (80 vs 79%, *p* = 0.993), disease-free (85 vs 80%, *p* = 0.404), and metastasis-free survival (85 vs 81%, *p* = 0.384) as shown in Figs. 1b, c, and d respectively.

## Discussion

Increased rates of marijuana use among individuals born after 1950 coupled with rising incidences of HPV related OPSCC within the past 20 years has led to theorization of an association between the two [19, 20]. Pooled data from 9 case-control study by the INHANCE consortium showed a positive agreement of marijuana use with OPSCC (*p* = 0.009) [17]. This data agreed with previous case-controlled studies by Gillison et al., and Zhang et al. [7, 21]. Specifically Gillison et al., showed a link between HPV positive OPSCC and marijuana user (*p* = 0.007), an association that was also seen within the INHANCE consortium data. Since the establishment of this positive association however, there remains a paucity of data surrounding the actual effect marijuana has on survival within this segregated patient population. We therefore sought to bridge this gap utilizing a case-matched cohort study.

Overall survival for our cohort of HPV positive OPSCC patients showed no statistically significant difference between marijuana users and non-users at 2-year (90% vs 80%) and at 5-year estimates (80% vs 72%, *p* = 0.400). This finding is even more pronounced in 2- (87% vs 86%) and 5-year disease specific survival estimates (80 vs 79%, *p* = 0.993). Disease-free survival (*p* = 0.404) and metastasis-free survival (*p* = 0.384) showed similar findings at 5 years where there was no statistically significant difference between the two cohorts. The finding of similar survival outcomes between the two cohorts seems to support theories from previous studies that suggests riskier sexual activity was the ultimate cause of increase HPV-related OPSCC within the marijuana user cohort [7, 17]. Within our cohort of patients, we were able to case match based on HPV status and OPSCC subsite as well as other factors, which coupled with the survival similarity between marijuana users and non-users suggested the lack of any survival benefit from no marijuana use.

Our observation of age, gender, and cT-Stage as predictors of disease-recurrence is consistent with other numerous findings within the literature for HPV-related OPSCC [10, 22]. Treatment modality within our cohort of patients consisted of a small subset of up-front TORS patients within the marijuana (12.8%) and non-marijuana user groups (12.7%) who were found to have no statistically significant difference in predicting disease-recurrence (*p* = 0.787). This is fitting within the literature where survival outcomes of TORS and primary chemotherapy and/or radiotherapy were found to have similar results [23–25]. Selection of TORS patients within our institution was stringent and as a result may represent a patient selection confounder. However, this finding certainly supports an increasing body of literature that shows TORS has similar oncologic outcomes with potential benefit in functional outcomes compared to patients treated with chemoradiotherapy when patients are selected appropriately [26, 27]. Most interestingly, the finding of no prognostication from cN-Stage on disease recurrence fits in within a growing body of literature that have shown high nodal number rather than cN-staging as a predictor of recurrence within the HPV-related OPSCC patient population [28, 29]. This finding was found within both cohorts of marijuana users and non-users, supporting again the lack of an oncologic outcome difference between the two groups.

Limitations of this study are acknowledged. The case-matched design of the study precluded any ability to definitively delineate any cause and effect between marijuana and OPSCC. In addition, marijuana users were not able to be classified into amount of marijuana used, specific forms of marijuana used, or years of usage due to limitations in patient numbers and data collection. As a result, true quantification of effect owing to length and amount of marijuana use couldn’t be analyzed. Despite that, owing to the case-matched design of the study as well as the largely prospective collected database, we were able to control and match for age, gender, cT-stage, as well as cN-stage. Nevertheless, it is highly unlikely that any randomized trial will address this question and further clarification of the risk of marijuana use will likely only come from larger retrospective cohorts.

The results of this study found no evidence that marijuana results in adverse outcomes within an HPV-related OPSCC patient population. Overall, disease-specific, disease-free, as well as metastasis free survivals were not found be significantly different between marijuana users and non-users. In addition, age, gender, and cT-stage but not cN-Stage or treatment modality were predictive of disease recurrence within both patient cohorts.

This study also suggests that future use of marijuana as an anxiolytic and analgesic within the HNC population may be safe, without significant concern for detrimental outcomes.

## Conclusion

No survival difference is found between marijuana users and non-users within HPV-related OPSCC patients. This study did not reveal evidence that marijuana leads to adverse events in HPV related OPSCC patients. However, future prospective studies with larger sample sizes should be performed to validate these findings.

## Data Availability

The datasets used and/or analyzed during the current study are available from the corresponding author on reasonable request.

## Abbreviations

CCI: Charlson comorbidity index
CRT: Chemoradiation therapy
HNC: Head and neck mucosal cancer
HPV: Human papillomavirus
OPSCC: Oropharyngeal squamous cell carcinoma
TORS: Transoral robotic surgery
